# Supplementary material for: Determinants of knowledge on sexually transmitted infections among students in public higher education institutions in Melaka state, Malaysia
Source: PLoS One. 2020 Oct 29;15(10):e0240842. doi: 10.1371/journal.pone.0240842 (PMC7595423; doi:10.1371/journal.pone.0240842)
Supplement: Attachment — Submitted filename: Response to reviewer-29September2020.docx [file pone.0240842.s002.docx]

**Response to Reviewers.**

Reviewer #1: All comments are addressed, and no further corrections are required. However, paper may get more clarity upon getting English editing.

Thank you for your comment: We have sent for English proofread. The amendments are highlighted by the track changes.

Reviewer #5: Review manuscript PONE-D-20-11879R1
“Determinants of knowledge on sexually transmitted infections among students in public higher education institutions in Melaka state, Malaysia".

The authors have successfully addressed all reviewers’ comment.
I now have only few minor comments/editing points as follows;
-Page 7 line 166, there should be a period after the close parenthesis.

Thank you for your comment. We have addressed it. See Page 7, line 172.

-Page 7 line 169, the letter “s” should be removed.

Thank you for your comment. We have addressed it. See Page 8, line 175.

-Page 8, the Table 1 should have a bottom line.

Thank you for your comment. We have addressed it. See Page 8, Table, 1.

-Page 13 There are several unclear terms in the Table 3 that might require clarification.
“having sex man with man” should be either “homosexual relationship” or “same sex relationship”
“Abnormal discharges” should be accompanying with the organ where it came from i.e. vagina, nasal, or urethral.
“Be faithful” should be written in a full term as “Be faithful in the intimate relationship”

Thank you for your comment. We have addressed these. See Page 13, Table 3.

-Please specify the full term of abbreviations used in the Tables in each footnote, to make them easier for readers to read and understand at a glance.

Thank you for your comment. We have addressed these. See Page 8, Table 1. Page 12, Table 2. Page 14, Table 3. Page 14, Table 4. Page 15, Table 5. Page 16, Table 6.
